# Inappropriate medication use and risk of falls – A prospective study in a large community-dwelling elderly cohort

**DOI:** 10.1186/1471-2318-9-30

**Published:** 2009-07-23

**Authors:** Sarah Berdot, Marion Bertrand, Jean-François Dartigues, Annie Fourrier, Béatrice Tavernier, Karen Ritchie, Annick Alpérovitch

**Affiliations:** 1 INSERM, U708, Paris, France; 2 Univ Pierre et Marie Curie-Paris 6, Paris, France; 3 INSERM U897, ISPED-Victor Segalen University, Bordeaux, France; 4 INSERM U657, Victor Segalen University, Bordeaux, France; 5 Department of Geriatrics, CHU de Dijon, Dijon, France; 6 INSERM U888, Hôpital La Colombière, Montpellier, France

## Abstract

**Background:**

Explicit criteria for determining potentially inappropriate medication consumption in elderly were elaborated by Beers et al. These lists have been used worldwide to evaluate medical prescriptions but there is little epidemiologic evidence demonstrating negative consequences of inappropriate medication use. It has been reported that some drugs could increase the risk of falls, which are a frequent and serious problem in elderly population. We aimed to evaluate the association between the use of potentially inappropriate medications and the risk of falls.

**Methods:**

The 3C Study is a multicentre prospective cohort study conducted in France with 4 years of follow-up. Non-institutionalized men and women aged 65 years or over (N = 6343) were randomly selected from electoral rolls. Data on socio-demographic, medical characteristics and medication use (based on self-reports and data from the national healthcare insurance) were collected. Use of inappropriate medication for elderly was defined from established criteria. Data about falls were collected at the two follow-up examinations (2 years and 4 years after baseline). The association between the exposure to inappropriate medications and the risk of falls was evaluated using multivariate models (Cox model and logistic regression).

**Results:**

32% of subjects reported inappropriate medication use at baseline and 29% at least two of the three examinations; 22% had fallen 2 times or more during follow-up. Overall, inappropriate medication users had an increased risk of falling. This increase was mainly due to the use of long-acting benzodiazepines (adjusted odds ratio (OR) = 1.4, 95% confidence interval: [1.1–1.8], in both occasional and regular users), other inappropriate psychotropics (adjusted OR = 1.7 [1.7–2.7] in regular users), or medication with anticholinergic properties (adjusted OR = 1.6 [1.2–2.1] in regular users). Neither occasional, nor regular use of short- or intermediate-acting benzodiazepines was associated with an increased risk of falling. Further analysis in long-acting benzodiazepines users did not show any dose-effect relation between the number of prescriptions filled over a 3-year period and the risk of falling.

**Conclusion:**

Our study showed that use of inappropriate medications was associated with an increased risk of falling in elderly persons. This increase was mainly due to long-acting benzodiazepines and other inappropriate psychotropics, and to medications with anticholinergic properties.

## Background

In the United States, more than one-third of all medications is consumed by persons aged 65 years and over, although they only represent approximately 15% of the total population [1]. Similar data have been reported in other developed countries. Due to concurrent prescription of several drugs, the risk of inappropriate drug combinations is increased in older persons [2,3]. Moreover, medication metabolism is affected by aging-related pharmacokinetic and pharmacodynamic changes which increase both drug half-life and drug free fraction. Lastly, coexisting illnesses can interact with medications. For all these reasons, older persons are at higher risk of experiencing adverse drug effects. To reduce frequency and severity of these events is a current challenge for public health agencies.

Beers et al [4] have elaborated explicit criteria for determining potentially inappropriate medication consumption in older adults, using Delphi methods for obtaining a consensus of recognized experts. The Beers list, which initially defined inappropriate medication use in nursing home residents, was afterwards modified and updated for being used in general elderly populations [5-8]. The lists identified specific drugs or drug prescriptions (excessive dose, excessive treatment duration, inappropriate drug combination and coexisting illness) with unfavorable benefit/risk ratio or questionable efficacy. Inappropriate medication lists have been used worldwide for evaluation of medical prescriptions [9-13] but there has been little evidence from epidemiological studies regarding the negative consequences of inappropriate medication use [14-17].

Previous studies have suggested that some drugs could increase the risk of falls, which are a frequent and serious problem in elderly population [18-24]. In a one-year follow-up study of persons aged 75 years and over living in the community, about one-third reported at least one fall [25]; higher annual risk of falls, up to 50%, were reported in the oldest old or in persons living in nursing homes. The consequences of injuries and fractures due to falls (mortality, hospitalizations, disability, and institutionalization) increase with age. In 2001, the American and British 'Guidelines for the prevention of falls in older persons' therefore recommended careful review of medications taken by elderly persons [26].

The aim of this prospective study was to evaluate the association between the use of potentially inappropriate medications and the risk of falls in a large population-based cohort of elderly persons.

## Methods

### Study population

The 3C Study is a multicentre prospective cohort study conducted in France. Its primary objective is to evaluate the relationship between vascular factors and dementia. The detailed study protocol has already been reported elsewhere [27]. Briefly, non-institutionalized men and women aged 65 years or over were randomly selected from electoral rolls of three French cities. The acceptance rate was 37%. A total of 9294 people were included in the study (4931 from Dijon, 2104 from Bordeaux and 2259 from Montpellier). The present study uses data collected at baseline (1999–2000) and at the 2-year (2001–2002) and 4-year (2003–2004) follow-up examinations.

The study protocol was approved by the Ethical Committee of the University hospital of Kremlin-Bicêtre. Each participant signed legal consent forms.

### General data

Data were collected by trained nurses and psychologists during face-to-face interviews, using standardized questionnaires. For the purpose of the present study, the following baseline variables have been selected: socio-demographic characteristics (gender, age, educational level, living alone), past medical history, global cognitive performance, depressive symptoms, diurnal drowsiness, impaired mobility and body mass index (computed from height and weight measurements). The Mini Mental State Examination (MMSE) score, ranging from 0 to 30 (best score), was used as a measure of global cognitive functioning. Depressive symptoms were evaluated with the Centre for Epidemiologic Studies-Depression scale (CES-D). It consists of 20 self-report items about the frequency of depressive symptoms over the last week. Impaired mobility was assessed by three items of the Rosow and Breslau scale: doing heavy housework, walking half a mile and going up and down to the second floor [28,29]. Diurnal drowsiness was assessed in a questionnaire about sleep disorders.

### Measurement of exposure to potentially inappropriate medications

At baseline and follow-up examinations, the questionnaire included an inventory of all drugs used during the preceding month. To reduce underreporting, participants were asked to provide medical prescriptions, drug packages and any other relevant material. The names of the drugs were systematically coded using the Anatomical Therapeutic Chemical (ATC) classification system [30]. No data on dose, duration of treatment or reason of prescribing were collected. To cover the whole study period (1999–2004) and to take into account changes in drug marketing (new drugs and removals), we combined different lists of inappropriate medication [31,32]. Due to lack of information in the 3C study, criteria based on dose, treatment duration and reason for prescribing could not be considered.

The full list of criteria used to define inappropriate prescription is given in Table 1 (see Additional file 1). As the questionable efficacy of cerebral vasodilators was the main reason for considering these medications as inappropriate, we also used a list of inappropriate medications without this item. In addition, we built composite criteria combining several individual criteria to investigate further the impact of potentially inappropriate psychotropic drugs (anticholinergic antidepressants, antipsychotic drugs and anticholinergic hypnotic drugs) and potentially inappropriate medication with anticholinergic properties. Use of short- or intermediate-acting benzodiazepines in older persons is considered as inappropriate when dosage exceeds half the dose given to younger subjects. As information about dosage was not available, we could not identify inappropriate use of these drugs and we assumed conservatively that subjects under short- or intermediate-acting benzodiazepines took the appropriate dose.

**Table 1:** List of Potentially Inappropriate Medications Used in the Present Study*

**Criteria**	
Indomethacin	Cimetidine
Phenylbutazone	Stimulant laxatives
Concomitant use of 2 or more NSAIDs	Long-acting sulfonylureas
Anticholinergic antidepressants	Methocarbamol, baclofen, tetrazepam
Antipsychotic drugs	Cerebral vasodilators (dihydroergocristine, ginkgo-biloba, pentoxifylline, ...)
Anticholinergic hypnotic drugs	Meprobamate
Anticholinergic antihistamines	Gastrointestinal antispasmodic drugs with anticholinergic properties
Anticholinergic muscle relaxants and antispasmodic drugs	Antiemetics, cough suppressants, nasal decongestants, or antidrowsiness drugs with anticholinergic properties
Concomitant use of drugs with anticholinergic properties	Dipyridamole
Long-acting benzodiazepines (half-life ≥ 20 h)	Nitrofurantoin
Centrally acting antihypertensives	Concomitant use of 2 or more psychotropic drugs from the same therapeutic class
Short-acting calcium-channel blockers	Concomitant use of anticholinesterase drugs and drugs with anticholinergic properties
Reserpine	Barbiturates (except phenobarbital)
Disopyramide	Doxazosin
Ticlopidine	

* According to the lists proposed by Beers [5], Fick [6] and Laroche [7].

Exposure to inappropriate medication was measured in two ways. The first exposure measurement was only based on baseline data. We created a binary variable to define the overall exposure to at least one of the criteria listed in table 1 and a series of binary variables for examining the exposure to each individual criterion. Then, using data collected at both baseline and the two follow-up examinations, exposure to inappropriate medication was categorized as: never user of any inappropriate medication (reference group), user of inappropriate medication (any or individual item) at one examination (occasional user) and user at two or three examinations (regular user). The total number of other drugs used (i.e. excluding inappropriate medication) was also computed.

About half of the 3C study participants were affiliated to the French national healthcare insurance for active or retired salaried workers (Caisse Nationale d'Assurance Maladie des Travailleurs Salariés (CNAM-TS)). For these persons, we obtained from the CNAM-TS individual data about all drugs prescribed between 2001 and 2003. In France, psychotropic drugs cannot be obtained without medical prescription. The delivery of psychotropic medication to patients cannot exceed the quantity necessary for one-month treatment, so patients under long-duration treatment are required to buy medications monthly. We used the healthcare insurance data for assessing further psychotropic use. For both long-acting and short- or intermediate-acting benzodiazepines, we computed the number of boxes bought between 2001 and 2003, as registered in the CNAM database. This number was used as a quantitative measure of the exposure. For further analysis, this variable was categorized for comparing heavy users to subjects with lower consumption of benzodiazepines

### Falls

At baseline and at each follow-up examination, participants were asked about the occurrence of falls. No precise definition of a fall was given to the participant before the question being asked. The baseline question ("Did you fall in the preceding months?") did not refer to a precise number of months, while the follow-up question ("Did you fall since the preceding examination?") covered a 2-year period. Participants who reported to be fallen were asked about the number of falls. Precise dates of falls were not collected. The number of falls during follow-up (incident falls) was categorized as no fall, one fall, two or more falls.

### Data analysis

Among the initial 3C study population (9294 subjects), 2178 were excluded because they had diseases which might affect gait and equilibrium (stroke (n = 291) and Parkinson's disease (n = 73)), had fallen before baseline evaluation (n = 1656), or both (n = 158). Of the remaining 7116 subjects, further 773 subjects (10.9%) for whom no data about falls had been collected during the follow-up were also excluded. These subjects consumed more inappropriate medication at baseline (37.9%, p < .001). Our study population was finally composed of 6343 individuals.

The baseline characteristics of the study population were categorized as follows. Education level was classified according to the number of years of schooling: 5 years or less, 6–9 years, 10–12 years and more than 12 years. The body mass index was used as a three-category variable: normal (< 25), excess weight (25–29), obesity (> 29). Participants were classified as having impaired mobility if they reported impairment for at least one of the three items of the Rosow and Breslau scale. According to the French validation of the CES-D scale, women scoring over 22 and men scoring over 16 were considered to have depressive symptoms. Participants having a MMSE score higher than 27 were considered to have good cognitive functioning. After having excluded inappropriate medications, participants were categorized as using < 5 or ≥ 5 other drugs.

Associations between falls and the main characteristics of the participants were assessed with chi-square tests (see Additional file 2). The association between the exposure to inappropriate medications and the risk of falls was evaluated by computing risk ratios (RR) and their 95% confidence intervals (95% CI) using Cox regression models, except for comparing use of inappropriate medication in never, occasional and regular users. Indeed, for the latter comparison, we did not know whether longitudinal change in medication use, if any, had occurred before or after the fall. We therefore assessed the association by computing odds ratios and their 95% CI from logistic regression models. The following covariables were entered in the logistic regression models: age, gender, study centre, body mass index, diurnal drowsiness, depressive symptoms, cognitive functioning, impaired mobility and number of drugs used (excluding inappropriate medications). Further adjustment on use of other inappropriate medications (when examining a given class) was also performed. Interactions between inappropriate medication use and other variables were evaluated by testing the statistical significance (p < .05) of the interaction terms.

All statistical analyses were performed using SAS statistical software, release 9.1 (SAS Institute Inc, Cary, NC, USA).

## Results

### Characteristics of the study population

The baseline characteristics of the study population are shown in Table 2. The 6343 participants had a mean age of 73.7 years (SD: 5.3) and 59% were women. During the four years of follow-up, 42.1% of subjects had fallen at least once and 21.8% had fallen 2 times or more. The incidence of falls increased with age and was higher in women than in men. It was significantly increased in subjects with depressive symptoms, impaired mobility, and diurnal drowsiness. Consumption of 5 or more drugs (excluding inappropriate drugs) was also a risk factor of falls. Further analysis showed that, for most characteristics, subjects who did not report any fall did not differ significantly from those having fallen only one time, while the latter group differed from subjects who reported two falls or more. In the following analyses, the 'No fall' and 'Only one fall' groups were therefore put together.

**Table 2:** Number of Falls during the 4-year follow-up according to the Baseline Characteristics of the Study Population

**Variable n (%)**	**No fall (n = 3670)**	**One fall (n = 1291)**	**≥ 2 falls** **(n = 1382)**	**crude p-value**
Women	1922 (52)	833 (65)	987 (71)	<.001
Age (years)				
<75	2364 (64)	733 (57)	707 (51)	<.001
75–79	886 (24)	364 (28)	419 (30)	
≥80	420 (12)	194 (15)	256 (19)	
Living alone	1107 (30)	494 (38)	574 (41)	<.001
Years of schooling				
≤5	1204 (33)	420 (33)	474 (34)	.86
6–9	1086 (30)	378 (29)	400 (29)	
10–12	479 (13)	183 (14)	185 (14)	
≥13	901 (24)	308 (24)	322 (23)	
Body Mass Index				
<25	1701 (46)	641 (50)	648 (48)	.09
25–29	1489 (41)	478 (37)	524 (38)	
≥30	464 (13)	166 (13)	196 (14)	
Depressive symptoms*	369 (10)	176 (14)	201 (15)	<.001
MMSE ≥ 28†	2117 (58)	748 (58)	762 (55)	.24
Impaired mobility‡	1351 (37)	579 (46)	724 (53)	<.001
Diurnal drowsiness	554 (16)	206 (17)	259 (21)	.001
Number of drugs used ≥ 5 §	1140 (31)	459 (36)	573 (41)	<.001
At least one inappropriate medication	1092 (30)	408 (32)	504 (36)	<.001

* Centre for Epidemiological Studies-Depression scale: women scoring over 22 and men scoring over 16 were considered to have depressive symptoms.

† Mini Mental State Examination score.

‡ Participants were classified as having impaired mobility if they were dependant for at least one activity of the Rosow and Breslau scale.

§Inappropriate medications excluded

About one-third (31.6%) of the 3C study participants reported using at least one inappropriate medication at study entry; only 8% used two or more inappropriate medications. The overall proportion of users was significantly associated with the occurrence of falls during follow-up (Table 2). Cerebral vasodilators were the most frequently reported item (16.3%); the proportion of inappropriate medication users was 19.5% when this item was excluded. Other criteria were reported by less than 5% of the participants, except long-acting benzodiazepines (7.8%) and inappropriate medication with anticholinergic properties (5.0%). Inappropriate psychotropic drugs other than long-acting benzodiazepines were consumed by 2.1% of the participants. In addition, short- or intermediate-acting benzodiazepines were used by 12.2% of participants.

### Association between baseline use of inappropriate medication during the 4 years and risk of falls

The association between baseline use of at least one inappropriate medication (including or not including cerebral vasodilators) and risk of falls did not remain significant in fully adjusted Cox regression models (Table 3); there was neither any increased risk of falls in participants using two or more inappropriate drugs (adjusted RR = 1.14 [0.95–1.37]). Analyses focusing on specific criteria showed that users of long-acting benzodiazepines had a significantly increased risk of falling two times or more during follow-up. The risk of falls was also slightly increased, but not significantly, in users of other psychotropic drugs inappropriate for elderly and in users of inappropriate medication with anticholinergic properties. Subjects using short- or intermediate-acting benzodiazepines were not at increased risk of falls. Further adjustment for use of other inappropriate medications did not change these results.

**Table 3:** Association between Inappropriate Medication Use and Risk of Fall

	**≤ 1 fall**	**≥ 2 falls**				
**Medication n (%)**	**(N = 4961)**	**(N = 1382)**	**RR (95% CI)**	**crude P-value**	RR (95% CI)*	P-value*
Inappropriate medication, full list	1500 (30.2)	504 (36.5)				
Inappropriate medication, excluding cerebral vasodilators	924 (18.6)	315 (22.8)	1.27 (1.12–1.43)	<.001	1.05 (0.92–1.20)	.47
Long-acting benzodiazepines	351 (7.1)	144 (10.4)	1.46 (1.23–1.74)	<.001	1.20 (1.00–1.43)	.048
Inappropriate psychotropic drugs	108 (2.2)	47 (3.4)	1.60 (1.20–2.14)	.002	1.31 (0.97–1.76)	.08
Inappropriate medication with anticholinergic properties	223 (4.5)	92 (6.7)	1.50 (1.21–1.85)	<.001	1.18 (0.96–1.47)	.13
Short- or intermediate-half-life benzodiazepines	568 (11.5)	206 (14.9)	1.31 (1.13–1.51)	<.001	0.99 (0.85–1.16)	.89

* Adjusted for age, sex, study centre, body mass index, diurnal drowsiness, number of drugs (excluding inappropriate medication), cognitive functioning, depressive symptoms and impaired mobility.

Assessment exposure based on both baseline and follow-up data showed that 53.7% of participants did not report any inappropriate medication use at any of the three examinations, 17.1% reported inappropriate medication at one examination only (occasional users) and 29.2% at two or three examinations (regular users). The risk of falls was significantly increased in regular and/or occasional users of at least one inappropriate medication (full list or excluding cerebral vasodilators) (Table 4). Both occasional and regular users of long-acting benzodiazepines were at higher risk of falls than never users. For inappropriate psychotropics or medication with anticholinergic properties, odds ratios were significantly increased in regular users only. In contrast, neither occasional use nor regular use of short- or intermediate-acting benzodiazepines were associated with falls.

**Table 4:** Risk of Fall in Occasional and Regular Users of Inappropriate Medication compared to Never Users

	**≤ 1 fall**	**≥ 2 falls**				
**Medication n (%)**	**(N = 4961)**	**(N = 1382)**	**OR (95% CI)**	**crude P-Value**	OR (95% CI)*†	**P-Value**†
**Inappropriate medication full list**						
Occasional user‡	809 (16)	277 (20)	1.48 (1.26–1.74)	<.001	1.23 (1.04–1.45)	.016
Regular user §	1389 (28)	465 (34)	1.45 (1.26–1.66)	<.001	1.08 (0.94–1.25)	.29
**Inappropriate medication without cerebral vasodilators**						
Occasional user	704 (14)	239 (17)	1.38 (1.17–1.63)	<.001	1.22 (1.02–1.45)	.030
Regular user	826 (17)	300 (22)	1.48 (1.27–1.72)	<.001	1.19 (1.00–1.41)	.049
**Long-acting benzodiazepines**						
Occasional user	286 (9)	117 (13)	1.58 (1.26–1.98)	<.001	1.40 (1.10–1.79)	.006
Regular user	316 (9)	135 (15)	1.65 (1.33–2.04)	<.001	1.41 (1.12–1.79)	.004
**Inappropriate psychotropic drugs**						
Occasional user	102 (2)	37 (3)	1.33 (0.91–1.95)	.14	1.17 (0.74–1.83)	.50
Regular user	74 (1)	39 (3)	1.93 (1.30–2.86)	.001	1.74 (1.14–2.66)	.010
**Inappropriate medication with anticholinergic properties**						
Occasional user	264 (5)	91 (7)	1.29 (1.01–1.65)	.042	1.21 (0.93–1.58)	.15
Regular user	172 (3)	84 (6)	1.83 (1.40–2.40)	<.001	1.57 (1.18–2.10)	.002
**Short- or intermediate-half-life benzodiazepines**						
Occasional user	429 (9)	151 (11)	1.34 (1.10–1.63)	.004	1.20 (0.96–1.49)	.11
Regular user	441 (9)	153 (11)	1.32 (1.08–1.60)	.006	1.05 (0.83–1.32)	.69

* Reference group: Never user of inappropriate medication

‡ Occasional user: exposure at only one of the 3 examinations (baseline and 2 follow-up)

§Regular user: exposure at two or three examinations

† Adjusted for age, sex, study centre, Body Mass Index, diurnal drowsiness, number of drugs (excluding IM), cognitive functioning, depressive symptoms and impaired mobility.

**Figure 1:**
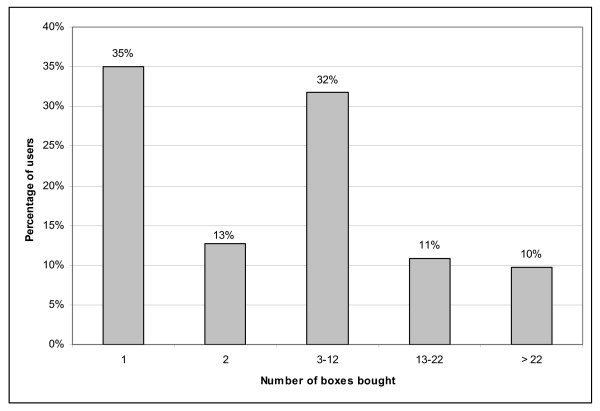
**Distribution of long-acting benzodiazepines users (N = 843) according to the number of boxes bought between 2001 and 2003**.

Data from the national healthcare insurance plan were obtained for 53% of the study population. Analysis focused on benzodiazepines. Over a 36-month period (2001–2003), 25.1% of the 3357 subjects bought at least one box of long-acting benzodiazepines and 34.5% at least one box of short- or intermediate-acting benzodiazepines. As shown on Figure 1, among the 843 long-acting benzodiazepines users, 35% bought only one box between 2001 and 2003, 50% less than two boxes, 20% more than 13 and 10% more than 22 boxes (last decile). Analysis did not show any dose-effect relation between the exposure to long-acting benzodiazepines, measured by the number of boxes, and the risk of falls. The risk of falls was only increased, but not significantly, in the 10% of the study population with the highest consumption (> 22 boxes) (adjusted OR = 1.35 [0.78–2.33]). Analysis detected a significant interaction between the level of exposure to long-acting benzodiazepines and the MMSE score (interaction: p = .02). Among subjects with lower MMSE score (< 28), those with the highest benzodiazepine consumption (> 22 boxes) had a significantly increased risk of falls (adjusted OR = 2.20 [1.07–4.51], p = .03), while no association between level of exposure and falls was found in participants with higher MMSE score. Among users of short- or intermediate-acting benzodiazepines, there was no increased risk of falls, whatever the level of exposure to these medications.

## Discussion

In this large community-based study, we found that use of inappropriate medications increased the risk of falls in elderly people. Consumption of long-acting benzodiazepines was responsible for the main part of this increase. But regular exposure to inappropriate psychotropic drugs or to inappropriate medication with anticholinergic properties was also associated with an increased risk of falling. Repeated assessments of medication use did not bring evidence for a dose-risk relation between the level or the duration of the exposure to inappropriate medications and the risk of falls. There was no association between use of short- or intermediate-acting benzodiazepines and occurrence of falls.

Overall, our results were consistent with those of previous studies about the relation between psychotropic medication and falls in older people [33-36]. However, previous studies, did not categorize psychotropic medications as appropriate or not appropriate for elderly and most of them did not differentiate benzodiazepines from other psychotropics. A few studies compared the risk of falls in long-acting and short- or intermediate-acting benzodiazepines users. Overall, unlike our study, they found that the risk of falls was independent of the half-life of the drug [37,38]. Tamblyn et al [39] found an association between falls and some specific benzodiazepines, but it was independent from their half-life.

The design of our multi-site study was well-suited to the exploration of the relation between drug use and falls, being a large general population-based cohort, with a high follow-up rate (92% at 4-year follow-up). Data relating to classical confounders were available and additional potential confounders, such as cognitive status, depressive symptoms, or diurnal drowsiness were assessed and taken into account in the statistical analysis. Drug consumption was assessed carefully. Compared with previous studies, one strength of the present study was the availability of measures allowing us to investigate the impact of prolonged and/or elevated exposure to inappropriate medication on the risk of falls. Lastly, we used recognized criteria for selecting medications that are considered as potentially inappropriate for elderly.

Some limitations of our study deserve, however, to be mentioned. Dosage and indication for prescription were not available. The precise date of falls was not collected. Data about some risk factors of falls, as visual acuity, were not available. Although our study population was quite large and the overall prevalence of inappropriate medication use was high (32%), [40-42] numbers were too small for estimating the risk of falls for specific drug classes, except for long-acting benzodiazepines. Another limitation which is common to all studies is that self-report of current drug use, as well as data on drug delivery from the health care insurance plan are imperfect measurements of actual drug consumption. Finally, our results may be conservative because subjects, whose data on falls were not available, consumed more inappropriate medication than the study population.

Most of the drugs potentially inappropriate for the elderly have known side-effects that can contribute to the risk of falling: drowsiness, decreased postural reflexes, extra-pyramidal symptoms, drugging and myorelaxant effect [43,44]. Age-related changes in drug metabolism and polypharmacy can increase the frequency and severity of these side effects, which in turn can increase the risk of falls. In the 3C study participants, the risk of falling associated with the use of inappropriate medication, although statistically significant, was moderate. We did not find any high-risk group that could be the target of possible interventions. However, a small-sized controlled intervention [45] in persons aged 65 years and over has shown a significant 66% decrease in the risk of falling after psychotropic medication withdrawal. Another argument in favour of such interventions is that a given drug can be associated with different health events. Intervention efficacy should not therefore be evaluated on a single end-point.

Our study showed that the risk of fall was moderately increased in elderly users of inappropriate psychotropics (long-acting benzodiazepines or others) compared to non users. However, under the hypothesis of a causal relationship, the number of falls attributable to the use of inappropriate drugs depends on the exposure prevalence. In countries with a high consumption of psychotropics, an important number of falls could be attributable to inappropriate use of psychotropics in elderly. Among European countries, France has a high consumption of psychotropic drugs, with about 50% of subjects over 70 years consuming psychotropics [46]. In a French elderly community-dwelling population, 31% subjects reported use of at least one benzodiazepine [47]. In Canada, 22.5% of elderly were prescribed benzodiazepines in 1998 [48].

Our results on the relation between the duration and/or intensity of the exposure to inappropriate medications and the risk of falls are complex. Overall, regular and occasional users of inappropriate medications had similar risk of falling. In long-acting benzodiazepine users, analysis did not show significant linear relation (nor even any trend) between the number of boxes bought over a 3-year period and the risk of falls, which was increased only in those who bought more than 22 boxes (last decile of the distribution). There are several non exclusive explanations for the absence of a dose-risk relationship. A first possible explanation is measurement errors. Available measurements (repeated self-reports of current use and data from the national healthcare insurance plan) are only surrogate markers of the biological exposure, which depends on actual drug consumption. Another explanation is a selection process. Given that persons experiencing drug side-effects are more likely to stop their treatment than those without any tolerance problems, it would follow that long-term users of inappropriate medication could be at lower risk of undesirable events. Another, and related, explanation could be that medication side-effects may be more pronounced at the beginning of the treatment than after some weeks of use. Under this hypothesis, the instantaneous risk of fall decreases when the treatment duration increases. In line with this explanation, a study [49] showed that the association between benzodiazepine use and falls was stronger within the 15 days following the treatment initiation than later on.

## Conclusion

Data from the 3C study showed that use of inappropriate psychotropic drugs, and particularly of long-acting benzodiazepines, was associated with an increased risk of falling in persons aged 65 years and over. Our study showed that short- or intermediate-acting benzodiazepines did not increase the risk of falls and, as recommended, they should be preferred to long-acting benzodiazepines in elderly patients.

## Competing interests

The Three-City study is conducted under a partnership agreement between the Institut National de la Santé et de la Recherche Médicale (INSERM), the Victor Segalen-Bordeaux II University, and Sanofi-Aventis. The Fondation pour la Recherche Médicale funded preparation and initiation of the study. The Three-City study is also supported by the following institutions: Caisse Nationale Maladie des Travailleurs Salariés, Direction Générale de la Santé, MGEN, Institut de la Longévité, Conseils Régionaux of Aquitaine and Bourgogne, Fondation de France, and Ministry of Research-INSERM Programme "Cohortes et collections de données biologiques".

Sarah Berdot was supported by a grant from the Fondation pour la Recherche Médicale.

The sponsors had no role in the design, methods, subject recruitment, data collection, analysis or preparation of the paper.

## Authors' contributions

SB performed the data analysis, interpretation of data and drafted the manuscript. MB performed the data analysis. JFD participated in the acquisition of subjects and data and helped to draft the final manuscript. KR participated in the acquisition of subjects and data and helped to draft the final manuscript. BT participated in the acquisition of data. AF participated in the acquisition of data. AA participated in the study concept, interpretation of the data and drafted the manuscript. All authors read and approved the final manuscript.

## Pre-publication history

The pre-publication history for this paper can be accessed here:



## Supplementary Material

Additional file 1 **Supplemental table 1**. List of All Drugs that Belong to Each Class of Criteria. Click here for file

Additional file 2 **Supplemental table 2**. Relation between the risk of falls and each variable included in the multivariate models. Click here for file
