# Supplementary material for: Inappropriate medication use and risk of falls – A prospective study in a large community-dwelling elderly cohort
Source: BMC Geriatr. 2009 Jul 23;9:30. doi: 10.1186/1471-2318-9-30 (PMC2721838; doi:10.1186/1471-2318-9-30)
Supplement: Additional file 1 — Supplemental table 1. List of All Drugs that Belong to Each Class of Criteria. [file 1471-2318-9-30-S1.doc]

**Additional file 1 : List of All Drugs that Belong to Each Class of Criteria**

| **Criteria** |
| --- |
| **Unfavourable benefit/risk ratio** |
| Analgesics |
| *Indomethacin* |
| *Phenylbutazone* |
| *Concomitant use of 2 or more NSAIDs* |
| Drugs with anticholinergic properties |
| *Anticholinergic antidepressants: clomipramine, amoxapine, amitriptyline, maprotiline, dosulepin, doxepin, trimipramine, imipramine* |
| *Antipsyychotic drugs: chlorpromazine, fluphenazine, propericiazine, levomepromazine, pipotiazine, cyamemazine, perphenazine* |
| *Anticholinergic hypnotic drugs: doxylamine, aceprometazine, alimemazine* |
| *Anticholinergic antihistamines: promethazine, mequitazine, alimemazine, carbinoxamine, hydroxyzine, brompheniramine, dexchlorpheniramine, dexchlorpheniramine-betamethasone, cyproheptadine* |
| *Anticholinergic muscle relaxants and antispasmodic drugs: oxybutynin, tolterodine, solifenacin* |
| *Concomitant use of drugs with anticholinergic properties* |
| Sedative or hypnotic drugs |
| *Long-acting benzodiazepines (half-life ≥ 20h: bromazepam, diazepam, chlordiazepoxide, prazepam, clobazam, nordazepam, loflazepate, nitrazepam, flunitrazepam, clorazepate, clorazepate-acepromazine, aceprometazine, estazolam)* |
| Antihypertensives |
| *Centrally acting antihypertensives: methyldopa, clonidine, moxonidine, rilmenidine, guanfacine* |
| *Short-acting calcium-channel blockers: nifedipine, nicardipine* |
| *Reserpine* |
| Antiarrhymthmics |
| *Disopyramide* |
| Antiplatelet drugs |
| *Ticlopidine* |
| Gastrointestinal drugs |
| *Cimetidine* |
| *Stimulant laxatives: bisacodyl, docusate, castor oil, sodium picosulfate, cascara, sennosides…* |
| Hypoglycaemic |
| *Long-acting sulfonylureas : carbutamide, glipizide* |
| Other muscle relaxants |
| *Methocarbamol, baclofen, tetrazepam* |
| **Questionable efficacy** |
| *Cerebral vasodilators: dihydroergocristine, dihydroergocryptine, dihydroergotxine, ginkgo-biloba, moxisylyte, naftidrofuryl, nicergoline, pentoxifylline, piracetam, piribedil, raubasine-dihydroergocristine, troxerutin-vincamine, vincamine, vincamine-rutoside* |
| **Unfavourable benefit/risk ratio and questionable efficacy** |
| Gastrointestinal drugs |
| *Meprobamate* |
| *Gastrointestinal antispasmodic drugswith anticholinergic properties: association with belladonna, clidinium bromure-chlordiazepoxide, dihexyverine, diphenoxylate-atropine, scopolamine, tiemonium* |
| Other drugs with anticholinergic properties |
| *Antiemetics, cough suppressants, nasal decongestants, or antidrowsiness drugs with anticholinergic properties : alizapdire, buclizine, dimenhydrinate, diphenhydramine, meclozine, metopimazine, oxomemazine, pheniramine, chlorphenamine* |
| Antiplatelet drugs |
| *Dipyridamole* |
| Antimicrobial |
| *Nitrofurantoin* |
| **Drug-drug associations** |
| *Concomitant use of 2 or more psychotropic drugs from the same therapeutic class* |
| *Concomitant use of anticholinesterase drugs with anticholinergic properties* |
| **Others** |
| *All barbiturates (except phenobarbital) except when used to control seizures, used as hypnotic or anxiolytic drugs* |
| *Doxazosin* |
