# Supplementary material for: Inappropriate medication use and risk of falls – A prospective study in a large community-dwelling elderly cohort
Source: BMC Geriatr. 2009 Jul 23;9:30. doi: 10.1186/1471-2318-9-30 (PMC2721838; doi:10.1186/1471-2318-9-30)
Supplement: Additional file 2 — Supplemental table 2. Relation between the risk of falls and each variable included in the multivariate models. [file 1471-2318-9-30-S2.doc]

**Additional file 2: Relation between the risk of falls and each variable included in the multivariate models**

| Variables n(%) | ≤ 1 fall (N = 4961) | ≥ 2 falls (N = 1382) | OR (95% CI) | crude P-value |
| --- | --- | --- | --- | --- |
|
| Women | 2755 (56) | 987 (71) | 2.00 (1.76-2.28) | <.001 |
| Age (years) |  |  |  |  |
| <75 | 3097 (62) | 707 (51) | 1 | . |
| 75-79 | 1250 (25) | 419 (30) | 1.47 (1.28-1.69) | <.001 |
| ≥80 | 614 (12) | 256 (19) | 1.83 (1.55-2.16) | <.001 |
| Study centre |  |  |  |  |
| Bordeaux | 1081 (22) | 335 (24) | 1.22 (1.06-1.42) | .008 |
| Dijon | 2721 (55) | 689 (50) | 1 | . |
| Montpellier | 1159 (23) | 358 (26) | 1.22 (1.06-1.41) | .007 |
| Body mass index |  |  |  |  |
| <25 | 2342 (47) | 648 (47) | 1.04 (0.91-1.18) | .57 |
| 25-39 | 1967 (40) | 524 (38) | 1 | . |
| ≥30 | 630 (13) | 196 (14) | 1.17 (0.97-1.41) | .10 |
| Depressive symptoms | 545 (11) | 201 (15) | 1.40 (1.17-1.66) | <.001 |
| MMS ≥ 28 | 2865 (58) | 762 (55) | 0.90 (0.80-1.02) | .09 |
| Impaired mobility | 1930 (39) | 724 (53) | 1.74 (1.54-1.97) | <.001 |
| Diurnal drowsiness | 760 (16) | 259 (21) | 1.33 (1.13-1.55) | <.001 |
| Number of drugs used ≥ 5 | 1599 (32) | 573 (41) | 1.49 (1.32-1.68) | <.001 |
